# Effects of Three-Dimensional Sodium Alginate Scaffold on Maturation and Developmental Gene Expressions in Fresh and Vitrified Preantral Follicles of Mice

**DOI:** 10.22074/IJFS.2020.134609

**Published:** 2021-06-22

**Authors:** Cyrus Jalili, Fuzieh Khani Hemmatabadi, Mehrdad Bakhtiyari, Amir Abdolmaleki, Fatemeh Moradi

**Affiliations:** 1 Medical Biology Research Center, Health Technology Institute, Kermanshah University of Medical Sciences, Kermanshah, Iran; 2 Anatomy Department, School of Medicine, Iran University of Medical Sciences, Tehran, Iran

**Keywords:** Oocyte Maturation, Sodium Alginate, Vitrification

## Abstract

**Background:**

Prior to chemotherapy interventions, *in vitro* maturation (IVM) of follicles through vitrification can be used to help young people conserve their fertility. The aim of study was to investigate effect of sodium alginat scaffold on follicles development and improvement of the culture medium.

**Materials and Methods:**

This experimental study was conducted on immature female BALB/c mice (12-14 days). Follicles were gathered mechanically and placed in α-Minimal Essential Medium (α-MEM) containing 5% fetal bovine serum (FBS). Some pre-antral follicles were frozen. The fresh and vitrified follicles were cultured in different concentrations of sodium alginate (0.25%, 0.5%, and 1%) and two dimensional (2D) medium for 12 days. The samples were evaluated for viability percentage, the number of MII-phase oocytes and reactive oxygen specious (ROS) level. Additionally, *Gdf9, Bmp15, Bmp7, Bmp4, Gpx, mnSOD* and *Gcs* gene expressions were assessed in the samples.

**Results:**

The highest and lowest percentages of follicle viability and maturation in the fresh and vitrified groups were respectively 0.5% concentration and 2D culture. There was no significant difference among the concentrations of 0.25% and 1%. Viability and maturation of follicles showed a significant increase in the fresh groups in comparison with the vitrified groups. ROS levels in the both fresh and vitrified groups with different concentrations of alginate showed a significant decrease compared to the control group. ROS levels in follicles showed a significant decrease in the fresh groups in comparison with the vitrified groups (P≤0.0001). The highest gene expression levels were observed in the 0.5% alginate (P≤0.0001). Moreover, the viability percentage, follicle maturation, and gene expression levels were higher in the fresh groups than the vitrified groups (P≤0.0001).

**Conclusion:**

Alginate hydrogel at a proper concentration of 5%, not only helps follicle get mature, but also promotes the expression of developmental genes and reduces the level of intracellular ROS. Follicular vitrification decreases quality of the follicles, which are partially compensated using a three dimensional (3D)

## Introduction

Beside the advances in the field of *in vitro* maturation (IVM) techniques, low applicable results are available. The IVM techniques can be used to help young people conserve their fertility throughout the vitrification of germ cells, before other therapeutic interventions like chemotherapy or radiotherapy. Over these procedures, the people lost their follicular reservoir partially or completely (1).

The modern technologies can help follicles grow in the culture medium and mature the oocyte for increasing fertility opportunity in patients under chemotherapy and radiotherapy procedures. These interventional methods cause full or partial destruction of the follicular reserve. 

In ovulation induction during assisted reproductive technology (ART), more follicles could grow, while the patient is at risk of ovarian hyperstimulation syndrome (OHSS). Conservation of follicles has critical medicinal applications.

The follicle vitrification is considered as a reliable method conserving follicles in the early stages of maturation. Since no ovarian stimulation is required in this approach, thus it can be used as an appropriate method for follicle conservation. Due to the small size, immature follicles are ideal for freezing (2). Ovarian reserve procedures have been developed to conserve the fertility potential in cancerous patients, during gonadotoxic treatments like chemotherapy or radiotherapy (3). Primary follicles can be obtained from fresh and vitrified ovarian tissues. They usually have a small oocyte arrested in the prophase stage of miosis. One proper way for conservation of ovarian follicles is known as the slow follicular freezing (4). Formation of ice crystals is a challenging difficulty in freezing process. The vitrification is currently introduced to overcome this issue (5). 

In addition to the oocyte physiological conditions, the culture medium as an impressive factor affects the quality of oocytes and embryos. Different types of culture systems have been invented, based on the follicle size and research purposes, to conserve and develop various degrees of follicular development and accompanied somatic cells (6). The low rate of viability and follicular maturation in two dimensional (2D) culture medium leads to application of three dimensional (3D) scaffold providing the proper follicular growth (7). The growth of follicle mainly depends on morphological changes of somatic and granulosa cells. Thus, any impairment in the morphology of follicular cells causes a delay in growth and incomplete development of preantral follicles, similar to those observed in the 2D culture system (8). The granulosa cells available in 2D culture of preantral follicles are scattered around the oocyte which disrupts the stimulation and feedback effects among the oocyte and granulosa cells. These cell communications have significant roles in transmission of both autocrine and/or paracrine signals (9). 3D porous polymer scaffolds as a substrate for cell growth and a provider for sufficient mechanical strength in order to cell survival maintenance are widely used in tissue engineering (10). According to the various studies, culture of preantral follicles with sodium alginate hydrogel can produce oocyte with a potential of high fertility rate (11). 

Alginate, like other 3D culture matrices, supply mechanical support for tissues. Nevertheless, the cellular proteins show no reaction with alginate matrix. Alginate hydrogels in culture media also support *in vitro* tissue growth (12). Thus, 3D culture medium conserves follicles more effectively, than the cell culture in flat plates (2D). Alginate is an applicable substrate for microencapsulation due to its biocompatibility, high water dependency and gel formation ability in the presence of sodium ions. This material contains alternant mannuronic acid chains producing tensile strength of gel. Thus, application of follicles without ovulation stimulation and IVM therapy are known as appropriate methods to recruit immature cells and prevent the OHSS.

Application of IVM for immature oocytes is an appropriate procedure for patients, especially those with polycystic ovary syndrome. IVM can reduce ovulation induction and risk of OHSS as one of the clinical emergencies. Thus, these artificial conditions in IVM medium must compensate to the inappropriate conditions available in the patient group. These created artificial environments are similar to that of the normal follicular conditions, which stimulate and mature the oocytes to grow and develop, while prevent the adverse effects of culture medium on the oocyte (13).

Although in many studies the impacts of different alginate concentrations on follicle development have been investigated, there is no study evaluating the effects of culture medium alterations on developmental potential of follicles, the consequences of different concentrations of 3D sodium alginate scaffold (0.25%, 0.5% and 1%) and vitrification on genomic changes of follicles. In the present study we aimed to simulate the development of preantral follicles in 3D culture medium prepared by various concentrations of sodium alginate. One of the crucial challenges is improvement of the culture medium. The purpose of this study was to investigate effect of culture medium changes on developmental potential of follicles as well as effect of different concentrations of 3D sodium alginate scaffold on genomic alterations of follicles.

### Medical treatment

### Animal groups and ovary preparation 

Eighty immature female BALB/c mice (12-14 days) with preantral follicles were grouped into eight (ten animals in each), including non-vitrified and warmed-vitrified groups. The mice were bred based on the 12 light/ dark photo-cycle at 23°C and 44% humidity. The animals were sacrificed by cervical dislocation. Following a longitudinal abdominal incision, the ovaries were dissected, the follicles were obtained mechanically and placed in α-Minimal Essential Medium (α-MEM, Aldrich Chemical Co., USA) containing 5% fetal bovine serum (FBS, Aldrich Chemical Co., USA). In order to reach the complete isolation of follicles, the additional tissues attached to the ovary were removed using an insulin syringe needle. It was then washed and incubated in culture medium (2). They were examined at different time points. All materials were obtained from the Sigma-Aldrich company (Aldrich Chemical Co., USA). Animals were handled based on the ethical guidelines of the Iran University of Medical Sciences (Tehran, Iran; Ethical permission number: IR.IUMS.REC.1395.9221313207).

### Isolation of preantral follicles

A G29 needle connecting to a 1 ml insulin syringe was used for follicles mechanical isolation by stereomicroscope. Normal preantral follicles with diameter of 100- 150 μm were detected as a central oocyte with bilayer granulosa cells.

### Preparation of sodium alginate hydrogel

All materials were provided by Sigma-Aldrich Company. To prepare alginate hydrogel, the sodium alginate at the concentrations of 0.25%, 0.5% and 1% was mixed with phosphate buffer saline (PBS). Then, 0.5 g of activated carbon was added to 1 g of sodium alginate powder to remove the alginate impurities. It was followed by filtration using a 0.26 μm Millipore Filter. Finally, it was kept at 4°C (14). Following washing the isolated follicles in culture medium, they were prepared for encapsulation in different concentrations of sodium alginate hydrogel. 

### Vitrification

The isolated follicles (100-150 μm in diameter) of vitrified-warmed group were washed in medium and transferred to an equilibration solution (pre-treatment) consisting of α-MEM medium, in addition 7.5% Ethylene Glycol (EG), 7.5% Dimethyl Sulfoxide (DMSO) and 10% FBS for 7 minutes. Then, they were transferred to a new vitrification solution, consisting of α-MEM medium with 15% DMSO, 15% EG, 0.5 M sucrose and 10% FBS for 3 minutes. All steps were performed at room temperature (22-24°C) using a stereomicroscope. An insulin syringe with a connector was also used to transfer the follicles into the vitrification straw as following 1 cm of vitrification solution (pulled into the syringe), immediately 0.5 cm of air, then 1 cm of vitrification solution containing follicles, followed ultimately by the air and vitrification solution. The vitrification straws were sealed using hematocrit sealant and immersed in a liquid nitrogen tank for a week (13).

### Warming

The straws taken out of the nitrogen tank were left at room temperature (24°C) for 10 seconds. They were cut by scissors and connected to a pulled-end insulin syringe. Contents of the straws were evacuated in a clean plate and transferred immediately to sucrose solutions under stereomicroscopy. 100 μl drops of sucrose solutions were put in a four-well plate. The follicles were placed in various concentrations of sodium alginate 1, 0.5 and 0.25 each and temperature 24°C for 5 minutes. All thawing solutions consisted of α-MEM medium and 10% FBS with descending different concentrations of sucrose, 1 M sucrose (thawing solution 1), 0.5 M sucrose (thawing solution 2), 0.25 M sucrose (thawing solution 3). The follicles were then incubated for 30 minutes in MEM-α medium containing 10% FBS and antibiotics (100 IU/ml penicillin, 100 μg/ml streptomycin and 0.25 μg/ml amphotericin B) (13).

### Encapsulation and 3D culture of preantral follicles

Initially, the preantral follicles were isolated from ovary and transferred separately into the 5 μl drop of sodium alginate with different concentrations. Then, they were transferred to a calcium bath (containing 140 mM of CaCl_2_ and 50 mM of NaCl) using a micropipette tip to establish calcium bonds and formulate hydrogel droplets encapsulated follicles. After 2 minutes, alginate hydrogel drops were collected from the calcium bath and rinsed in culture medium. The encapsulated follicles were evaluated by a microscope and only those follicles located in the center of hydrogel were gathered for culturing. Cultivation of the isolated follicles is crucial due to the nonvascular structure of granulosa cells. In this method, the follicles could be cultivated independently and checked for probable changes during cultivation. Each encapsulated follicle was transferred into 40 μl droplets of culture medium beneath the oil in a 96-well plate. In the previous approach, the α-MEM medium which was used contained 1% insulin transferrin selenite (ITS), 100 IU/ml penicillin, 100 μg/ml streptomycin, 100 IU/ml recombinant follicle stimulating hormone (rFSH), 5% FBS and 10 ng/ml recombinant epidermal growth factor (rEFF). Expire date of the culture medium was increased up to a week at 2-8°C in the refrigerator. The follicles were cultured in a humidified incubator for 12 days at 37°C and 5% CO_2_ . Half of the culture medium was changed every day (7). Trypan blue as vital stain was used to assess the viability of follicles and non-toxic effects of sodium alginate. For this purpose, several follicles in each replicate were selected randomly and stained with 0.3% trypan blue. The follicles were immersed in the dye for 30 seconds and then were observed under an inverted microscope following several washing processes. The cell membrane of dead cells changed into dark blue, but the living cells resisted against dye penetration and the cell membrane remained unstained. 

### Follicle retrieval

In order to perform RNA isolation and oocyte maturation, the encapsulated follicles with sodium alginate were removed from the hydrogel substrate on the day 12^th^. In 3D culture system, 5 mg of ethylene glycol tetraacetic acid (EGTA) was added to the culture medium to deplete the follicles from hydrogel. This process occurred for 5 minutes at 37°C in an incubator. For RNA isolation and other biochemical analyses, the obtained follicles were transferred into the -80°C refrigerator in sterile test tubes with a minimum amount of medium (7). The follicles required for maturation were transferred to a petri dish containing medium

### Ovulation of maturation induction and oocyte maturation

For induction of follicular ovulation, 10 ng/ml of epidermal growth factor (EGF) was mixed with medium containing 1.5 IU/ml human chorionic gonadotropin (hCG). They were suspended with 5% CO_2_ and 37°C for 18 hours. Next, 0.1% of hyaluronidase was used to separate the cumulus cells around the oocyte (7). The oocytes were aspirated and counted using a glass pipette and evaluated for maturation, germinal vesicle (GV), germinal vesicle breakdown (GVBD) and metaphase II (MII) by the inverted microscope. Finally, the count and percentage of matured oocytes were assessed.

### Evaluation of morphological changes and percentage of follicles viability

An inverted microscope was hired to assess the percentage of follicles viability. Thus, the follicles were examined morphologically at the end of day 12^th^ of culture. The follicles with following features were considered as degenerated: arrest in proliferation of granulosa cells, cessation in follicular growth, early ovulation and dark follicles.

### 2D culture of follicles

For the control group, the preantral follicles were separately transferred into the α-MEM medium immediately after removing ovarian tissue. This medium contained 10% FBS, 100 IU/ml penicillin, 100 μg/ml streptomycin and growth factors similar to that of 3D culture.

### Measurement of reactive oxygen specious in oocyte

Level of the biochemical reaction among H_2_ O_2_ and DCFDA was considered as the level of intracellular free radical. Concentration of the required DCFDA for measurement of H_2_ O_2_ in the oocyte was 10 μM. 50 μl of DCFDA solution (available on plates as droplets) were warmed up in incubator for 30 minutes. The oocytes were placed in droplets of DCFDA and incubated for 20 minutes at 37°C in a dark environment. Next, the oocytes were transferred to other plates for washing and removing the remaining DCFDA. Following the three times washing, the oocytes were transported on the slide with a minimum culture medium (7). The slides were examined using fluorescence microscopy. The reaction between H_2_ O_2_ and DCFDA was visible inside the oocyte, reflecting green light at 460 nm (Olympus fluorescence microscope, Japan). Captured images of oocytes were examined using Image J (1.46r, USA) software. The background color was removed using software tools, but the color intensity of the oocytes also dropped to the same degree to avoid the error. By selecting the oocytes margin utilizing the software, amount of the obtained pixels was determined.

### RNA isolation and real-time polymerase chain reaction

Following the IVM procedure, total RNA was extracted using TRIzol (TRI reagent, Sigma, UK). Optical density (OD) was determined using a spectrophotometer and RNA quantity of each sample was was also analyzed. cDNA was constructed using Super-script II kit (Fermentase, Germany) in which random hexamer primers were hired to synthesize the cDNA. It was explicitly linked to mRNA as a template and provided the possibility of RNA transcription by a reverse transcriptase enzyme in the presence of dNTP. Realtime polymerase chain reaction (PCR) process was performed in triplicate using cDNA prepared from the oocyte of different groups. In addition, we used primers including ROX dye and SYBER Green Biosystem reagent (Applied Biosystems, USA) as passive control for signal intensity. Real-time PCR procedure was performed in the ABI Prism 7300 Sequence Detector (Applied Biosystems). Amplification carried 45 cycles and the optimal reaction conditions were included activation of polymerase enzyme at 95°C for 10 minutes, each denaturation cycle at 95°C for 15 seconds, annealing and elongation steps at 60°C depending on the temperature of the primer for 60 seconds (7). In addition to the primers designed in this study, *β-actin* primer was used as an internal control for standardizing (Table 1). Total reaction was performed in 45 cycles and three different technical replicates for each group.

### Statistical analysis

In this study, all methods were performed in three biological replications. Data were analyzed using SPSS software (version 22). Changes in the viability percentage, MII oocyte, ROS level and gene expression levels were evaluated by One-Way Analysis of Variance (ANOVA) among the groups. Data were represented as mean ± standard deviation (SD) and the significant level was considered as less than 0.0001 (P≤0.0001)

**Table 1:** Primers used for real-time polymerase chain reaction



Gene (Mus musculus)	Primer sequence (5'-3')	Annealing temperature (°C)	Product size (bp)
**Bmp15**	F: CTGATTGAGACCAACGGGAG R: TGCCAGCTTTAACACAGTTTTC	60	181
**Bmp4**	F: GTAGTGCCATTCGGAGCG R: ATCAGCATTCGGTTACCAGG	58	114
**Bmp7**	F: CTCAACGCCATCTCTGTCC R: CATCGAAGATTTGGAAAGGTGTG	59	143
**Gcs**	F: GTACCTTGAACGAGTGGATGAG R: GGTGGGATTTTAAGCAGATGC	62	98
**Gdf9**	F: GTCACCTCTACAATACCGTCC R: CGATTTGAGCAAGTGTTCC	61	92
**Gpx**	F: AACCTGACATAGAAACCCTGC R: CAGTAATCACCAAGCCAATGC	59	130
**mnSOD**	F: GTGAACAACCTCAACGCCAC R: GCTGAAGAGCGACCTGAGTT	60	99
**Actb**	F: GATTACTGCTCTGGCTCCTAG R: GACTCATCGTACTCCTGCTTG	61	151


## Results

**Fig.1:**
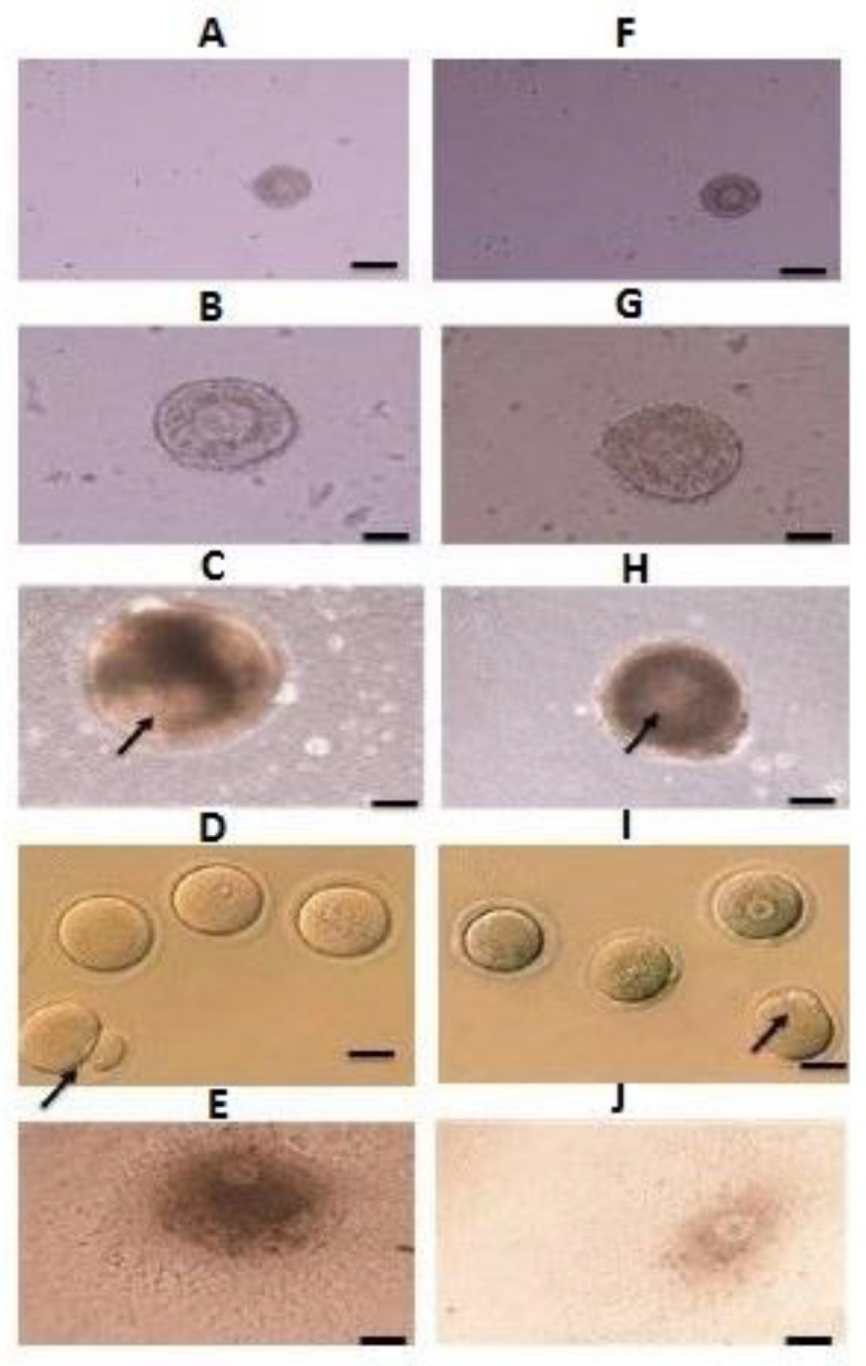
Fresh and vitrified follicles in 3D and 2D culture medium. **A-E.** Fresh, **F-J.** Vitrified follicles in **C, H. **3D, and **E, J. **2D culture medium. The arrows indicate in **C, H.** Antrum and **D, I.** Polar body. **A.** Day 1, **B.** Day 6, **C.** Day 12, **D.** MII- phase oocyte, **E. **Follicle in a 2D medium, **F. **Day 1, **G. **Day 6, **H.** Day 12, **I.** MII- phase oocyte and **J.** Follicle in a 2D culture medium (scale bar: 100 μm).

**Fig.2:**
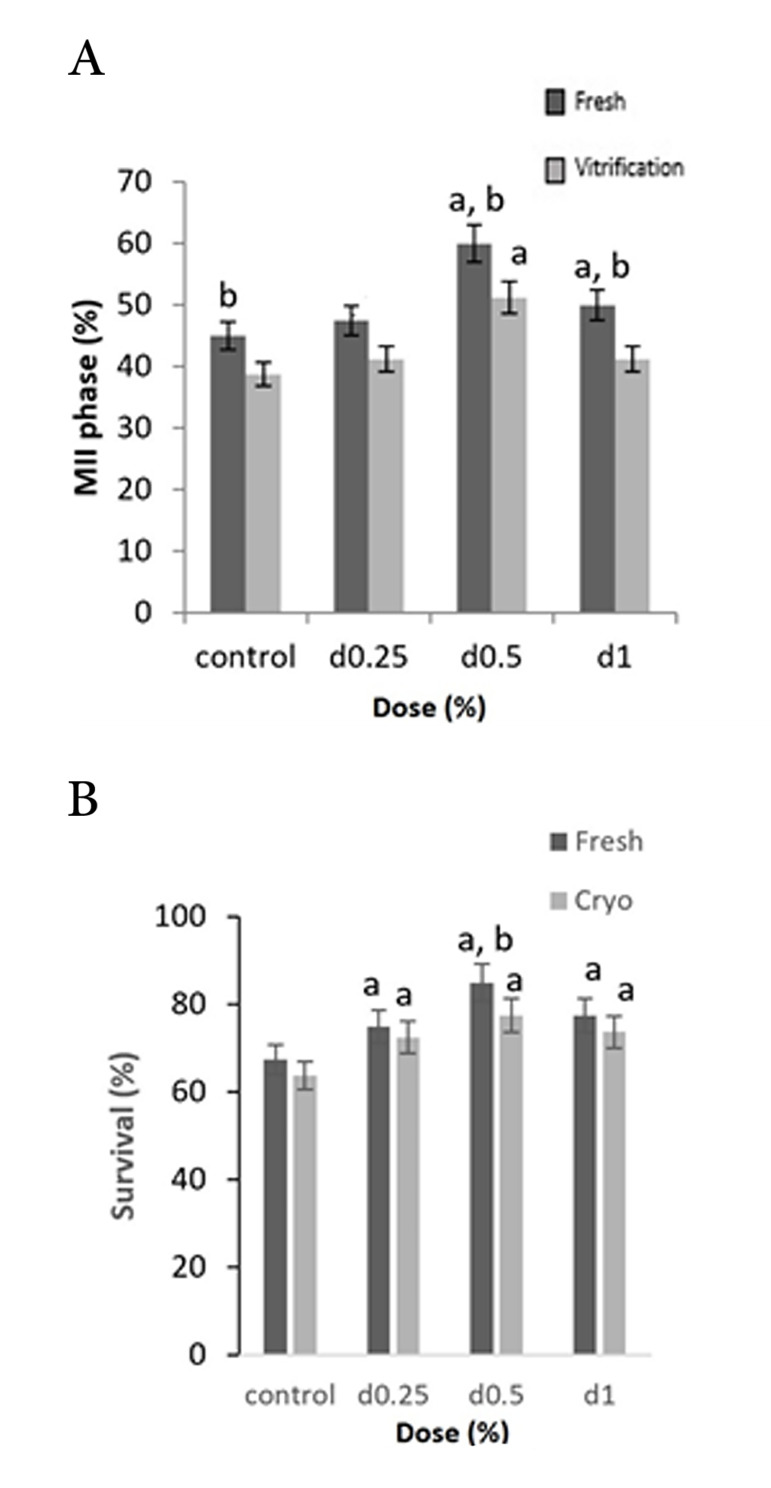
Various concentrations of 3D sodium alginate and 2D culture medium (control). **A.** Number of MII-phase oocytes and **B.** Viability percentage in the both fresh and vitrified groups. The highest viability percentage and MII-phase oocytes are related to the concentration of 0.5%. Number of MII-phase oocytes in the fresh groups is higher than the vitrified groups (P≤0.0001). a, b; Significant (P≤0.0001) difference than control and vitri- fied groups, respectively.

According to the Figure 1, the number of the survived follicles in both groups of fresh (n=60, 68, 62, Fig .1AE) and vitrification (n=58, 62, 59, Fig .1F-J) at different concentrations of sodium alginate was significantly increased, compared to the control group (n=54, 51, P≤0.0001). The concentration of 0.5% sodium alginate in comparison with the other concentrations was increased significantly, but there was no significant difference between the concentrations of 0.25% (n=60, 58) and 1% (n=62, 59). In the concentration of 0.5% (n=68), the survived follicles showed significant increase in the fresh groups in comparison with the vitrified groups (n=62), but this difference was not significant in the other groups (Table 2, Fig .2).

Comparison in follicles number of the fresh groups entered to the MII phase (Fig .2), indicating that the concentrations of 0.5% (n=48) and 1% (n=40) sodium alginate had a significant increase in comparison with the control group (n=32), but in the vitrified groups (Fig 2) only the concentrations of 0.5% (n=41) sodium alginate had a significant increase, in comparison with the control group (n=31, Fig .2); thus a significant increase in maturation level was observed in 3D culture medium (P≤0.0001). Moreover, comparison of maturation level at various concentrations of sodium alginate showed that the highest level of maturation was related to the concentration of 0.5% in the fresh (n=48) and vitrified (n=41) groups (Table 2). According to Figure 2, level of follicle maturation in all of concentrations of alginate as well as the control group was increased significantly (P≤0.0001) in the fresh groups compared to vitrified groups.

**Table 2:** Effects of various concentrations of 3D sodium alginate scaffolds (0.25%, 0.5% and 1%) and 2D culture medium (control) on survival, MII-phase oocytes, germinal vesicle breakdown (GVBD) and GV in the both fresh and vitrified groups


Groups	Alginate density (%)	Follicle (n)	Survival	MII	GVBD	GV

Fresh	0.25	80	60 (75)^*^	38 (47.5)	25 (31.25)	17 (21.25)
	0.5	80	68 (85)^*^	48 (60)^*^	22 (27.5)	10 (12.5)^*^
	1	80	62 (77.5)^*^	40 (50)^*^	26 (32.5)	14 (17.5)^*^
	Control	80	54 (67.5)	32 (45)	26 (32.5)	22 (27.5)
Vitrification	0.25	80	58 (72.5)^*^	33 (41.25)	22 (27.5)	15 (18.75)^*^
	0.5	80	62 (77.5)^*^	41 (51.25)^*^	25 (31.25)	14 (17.5)^*^
	1	80	59 (73.75)^*^	33 (41.25)	21 (26.25)	16 (20)^*^
	Control	80	51 (63.75)	31 (38.75)	20 (25)	29 (36.25)


Data are presented as n (%). The highest percentage of survival and MII-phase oocytes is related to the concentration of 0.5%. Number of MII-phase oocytes in the fresh groups is higher than in the vitrified groups. * ; Significant difference in comparison with the control group (P≤0.0001).

**Fig.3:**
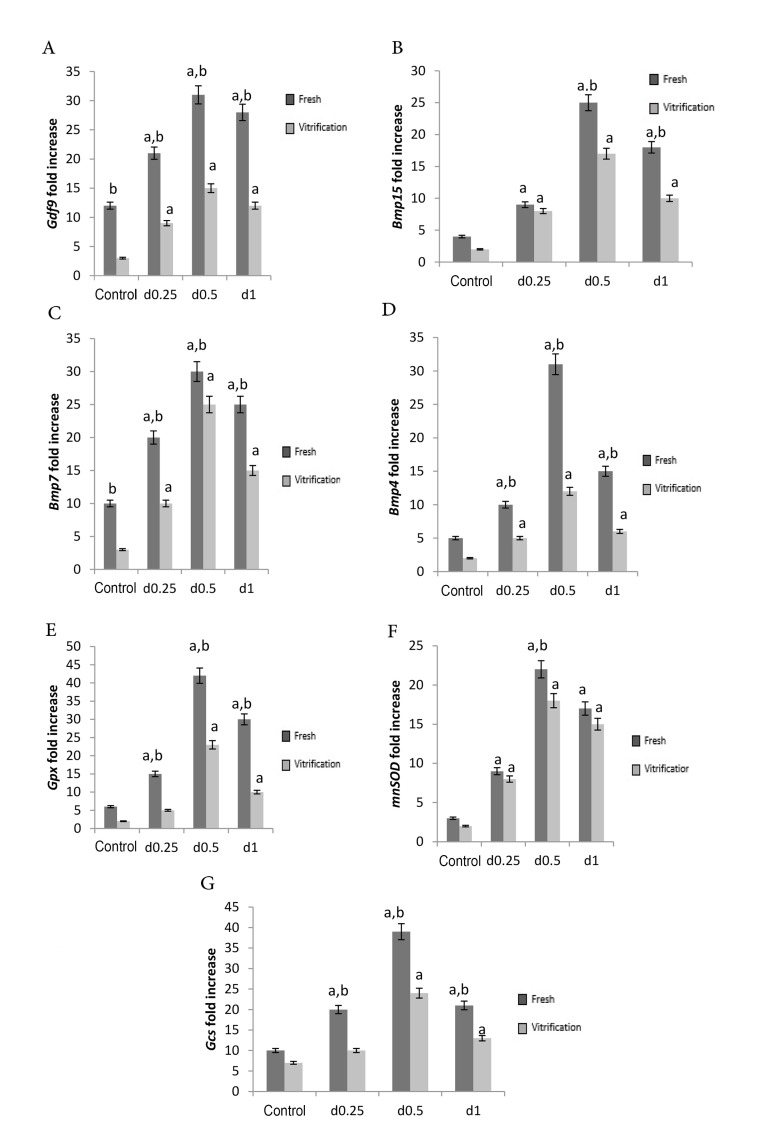
Different concentrations (0.25%, 0.5% and 1%) of 3D sodium alginate scaffolds and 2D culture medium (control) on genes expression. **A.** *Gdf9*, **B.** *Bmp15*,** C.*** Bmp7*, **D. ***Bmp4*, **E.** *Gpx*,** F. ***mnSOD*, and** G.** *Gcs*. Their expression level was more than the control group (P≤0.05). In both of the fresh and vitrified groups, the highest expression level of these genes was related to the concentration of 0.5% and the lowest was related to the control group. a; Significant difference than control and b; Significant difference than vitrified group (P≤0.0001).

The *Gdf9* gene expression level in the fresh groups was higher than that of the vitrified groups. In the both control and treatment groups, *Gdf9* gene expression level showed a significant increase in different concentrations of sodium alginate compared to the control group (P≤0.0001) and the highest expression level of this gene was related to the concentration of 0.5% (Fig .3).

In the both fresh and vitrified groups, *Bmp15* gene expression levels showed significant increase in all of the three sodium alginate concentrations, compared to the control group. In the fresh group, the highest *Bmp15* gene expression level was detected in the concentration of 0.5%. Expression level of this gene in 1% concentration was also higher than 0.25% (P≤0.0001). In the vitrified group, the highest expression level of Bmp15 gene was related to the concentration of 0.5% (P≤0.0001), but no significant difference was detected between the concentrations of 0.25% and 1%. In 0.5% and 1% of alginate, the expression level of this gene in the fresh group was higher than the vitrified group, but there was no significant difference between the fresh and vitrified groups at 0.25% concentration and control group (Fig .3).

The 3D sodium alginate scaffold showed positive and significant effects on Bmp7 expression in comparison with the control group (P≤0.0001). In the both fresh and vitrified groups, the lowest level of gene expression was related to the 2D culture medium and the highest expression level of this gene was observed in the concentration of 0.5%. *Bmp7* gene expression level in the fresh groups was higher than the vitrified group (Fig .3). In the both fresh and vitrified groups, *Bmp4* gene expression level was higher in all three concentrations of sodium alginate than in 2D culture medium. The highest expression level of this gene was observed in concentration of 0.5% (P≤0.001). Based on Figure 3, *Bmp4* gene expression level at different concentrations of sodium alginate was greater in the fresh group than the vitrified group (P≤0.0001). As it was shown in Figure 3, *Gpx* gene expression level showed a significant increase in all three concentrations of sodium alginate in the two fresh groups compared to the control group (P≤0.0001). The highest expression level of this gene was related to the concentration of 0.5%. *Gpx* gene expression level was higher in concentration of 0.1% than 0.25%. In accordance with Figure 3, *Gpx* gene expression level in all of the three concentrations of sodium alginate was higher in fresh groups than vitrified groups (P≤0.0001). In the both fresh and vitrified groups, the highest expression level of *mnSOD* gene was related to the concentration of 0.5% (P≤0.0001) and the lowest expression level of this gene was related to the 2D medium. The expression level of this gene was higher in the concentration of 0.1% rather than 0.25%. The expression level of this gene in the concentration of 0.5% in the fresh group was higher than that of the vitrified group, but in the other groups, no significant difference was found among the fresh and vitrified groups (Fig .3). *Gcs* gene expression level was significantly higher in the both fresh and vitrified groups with 0.5% concentration than the concentrations of 0.25% and 1% as well as the control groups. The lowest expression level of this gene was related to the control group. In the both fresh and vitrified groups, no significant difference was detected between the concentrations of 0.1% and 0.25%. Consistent with Figure 3, in all three concentrations of sodium alginate, *Gcs* gene expression level in the fresh group was significantly higher than that of the vitrified group (P≤0.0001). 

**Fig.4:**
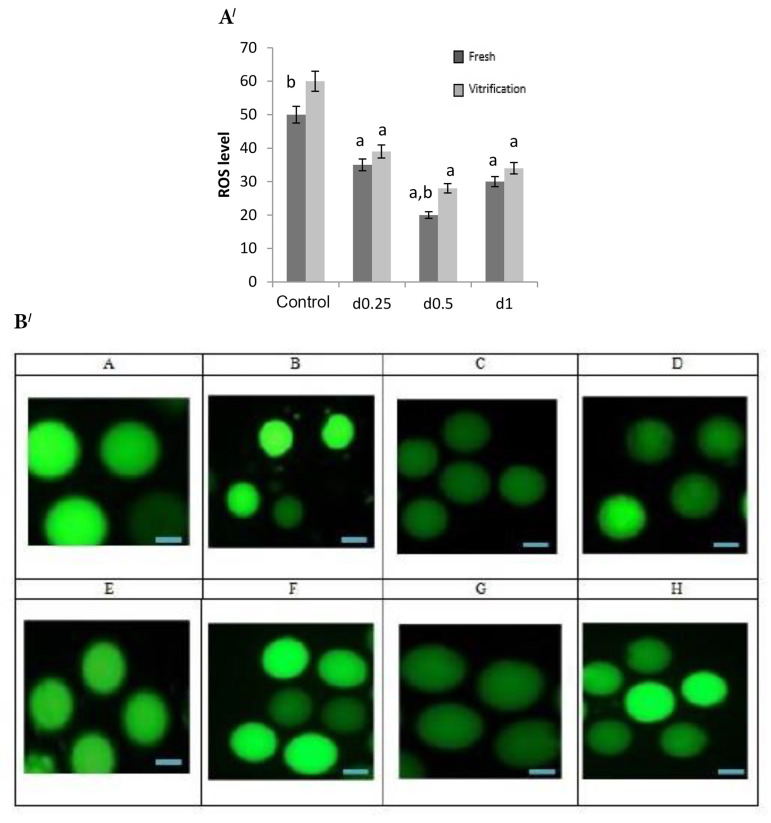
Effects of different concentrations of 2D and 3D culture medium on reactive oxygen specious (ROS) levels in both fresh and vitrified groups. **A/ .** Different concentrations (0.25%, 0.5% and 1%) of 3D sodium alginate scaffolds and 2D culture medium (control) on ROS levels; lowest ROS level is related to 0.5% sodium alginate and the highest ROS level is seen in control group. ROS level in 0.5% sodium alginate and 2D culture medium was lower in fresh group than vitrified group. a; Significant difference than control group, and b; Significant difference than vitrified group (P≤0.0001). **B/ . **ROS level in the fresh oocytes cultured in 3D sodium alginate medium; **A.** Control group,** B.** Concentration of 0.25%, **C.** Concentration of 0.5%, and **D.** Concentration of 1%. ROS level in vitrified oocytes cultured in 3D sodium alginate medium: **E.** Control group, **F. **Concentration of 0.25%,** G. **Concentration of 0.5%, and **H.** Concentration of 1%. The lowest ROS level was related to the concentration of 0.5% and the highest ROS level for control group (P≤0.0001, Scale bar: 100 μm).

According to the statistical results, level of free radicals in the fresh groups with 0.5% concentration showed a significant decrease compared to other concentrations and control group (P≤0.0001, Fig .4). The level of free radicals was decreased in both concentrations of 0.25% and 1%, compared to the control group, but there was no significant difference between the two concentrations of 0.25% and 1%. In the vitrified group, the highest free radical level was associated with the control group and the lowest level was related to 0.5% concentration. According to Figure 4, the level of free radicals in the concentration of 0.5% and 2D in the fresh group was lower than the vitrified group (P≤0.0001).

## Discussion

The results of present study approved a significant increase in the number of survived follicles in different concentrations of sodium alginate scaffold compared to the control group. According to the obtained results, both encapsulated and decapsulated follicles have not contributed to follicular damage and probably the encapsulated form of follicles can preserve the junctions between cells and the basement membrane of granulosa cells, leading to prevent follicular death (13). A significant increase was detected in the number of survived follicles encapsulated with 0.5% sodium alginate in the fresh groups compared to the vitrified group, but no significant alteration was found in the other concentrations between the other concentrations of sodium alginate in the fresh and vitrified groups. Filatov et al. (15) investigated the effects of vitrification and 0.1% alginate scaffold on follicle maturation. They concluded that 90% of the vitrified encapsulated follicles survived. This finding is in line with the results of our study containing 1% and 0.25% concentration groups as well as the control group. 

The hydrated 3D alginate meshwork provides specific conditions for cells to adhere, disperse, migrate and interact with the others. Thus, hydrogel is known as an excellent option for cell cultivation and differentiation in 3D culture medium. According to the findings of various studies, cultivation of preantral follicles in alginate hydrogel can provide survival of the high rate of in vitro follicles (16).

Follicular maturation is a process influenced by regulatory factors such as gonadotropin, secretory molecules, oocyte, surrounding granulosa cells and biological conditions of the oocyte itself (17). In IVM follicles, cytoplasmic and nuclear maturation occurs following the formation of metaphase II gametes. Significant advances have been made in developing *in vitro* gametogenesis (IVG) and maturation of follicles as well as the oocyte techniques in a wide range of mammalian species (18). Thus, sodium alginate is utilized as a 3D matrix for ovarian follicle encapsulation and maturation to produce the oocytes with fertility capability. This gel with unique biochemical properties is widely used in tissue engineering procedures and follicular culturing (2).

A comparison between the counts of MII phase follicles in the fresh group indicated that sodium alginate scaffold has significant effects on preantral follicle maturation compared to the 2D culture medium. Skory et al. (19) examined the growth of secondary follicles in alginate capsules with concentration of 0.5%. They concluded that 92% of follicles enter to MII phase. In the field of human follicles IVM, Lin et al. (4) concluded that the percentage of MII phase follicles in the 0.5% sodium alginate was decreased. This phenomenon implies that the follicles in humans and primates grow in the ovarian cortex, as an area with a high density of collagen. Thus, higher *in vitro* alginate concentration can provide an environment similar to that *in vivo* condition, resulting in increased levels of follicular maturation. However, due to the low collagen density in the ovary of mice, lower concentrations of alginate scaffold had more significant effects on follicle growth and gene expression. In murine follicles, reduction of alginate concentration in 3D scaffolds can improve the outcomes of IVM technique (4). Besides, the follicular maturation, in different concentrations of alginate, was significantly increased in the fresh groups compared to the vitrified groups. This outcome returns to the hydration-dehydration process during removal of antifreeze in preantral follicles exposed to vitrified-warmed process, which can induce changes in morphological features and survival rate of follicles. Long-term application of vitrification solution for follicles with leads to reduction of viability based on the toxic properties of antifreeze. In presence of the cells limited number, follicular and oocyte survival rates following vitrification is considered as an initial effect of vitrification.

IVM can change levels of gene expression, structure of mitotic spindles and metabolism of oocytes (20). The highest and lowest *Gdf9* gene expression level was related to the 0.5% concentration and 2D culture medium, respectively. Growth differentiation factor 9 (GDF9), through a direct effect on granulosa cells, induces rapid growth of follicles (21). Expression level of *Gdf9* gene in the fresh groups was higher than that of the vitrified groups. *Gdf9* is an ovulation agent strongly expressed in oocytes with a major effect on surrounding cells, especially granulosa, cumulus and theca cells. Paracrine interactions between the growing oocyte and surrounding cells are essential for both oocyte and follicular maturation (22). Song et al. (23) examined *Gdf9* gene expression level after 10 days follicular culture. They concluded that *in vitro* expression level of *Gdf9* gene was similar to the in vivo condition. In the both fresh and vitrified groups of the present study, *Bmp15* gene expression level showed a significant increase in all of the three concentrations of sodium alginate, compared to the control group and the highest *Bmp15* level was related to the concentration of 0.5% sodium alginate. BMP15 is a paracrine signaling molecule that interferes with growth of oocytes and the follicles. This protein may be involved in maturation, ovulation, follicular growth, regulation of the sensitivity of granulosa cells to follicle stimulating hormone (FSH), determination of the number of ovulating oocytes, prevention of apoptosis in granulosa cells and acceleration of oocyte maturation (24). Both of *Gdf9* and *Bmp15* firstly affect function of granulosa cells and then the oocyte itself (25). 

Parrish et al. (26) examined expression level of *Bmp15* and *Gdf9* genes *in vivo* and 0.25% alginate concentrated 3D culture medium. They reported that expression level of these two genes showed no significant difference in medium and 3D alginate culture in the stage of bilayered transition to multilayered secondary follicles. This finding confirmed effectiveness of 3D alginate scaffolds to stimulate physiological environment similar to that of the body to express developmental genes correctly. Expression level of *Bmp7* gene in different concentrations of sodium alginate in both of the fresh and vitrified groups showed that 3D sodium alginate scaffold has significantly positive effect on *Bmp7* expression in comparison with the control group. In the both fresh and vitrified groups, the lowest expression level of gene was related to the 2D culture medium and the highest level was seen in 0.5% concentration. *Bmp7* plays a vital role in the transition of primordial follicles to primary, preantral and antral follicles. *Bmp7* is secreted by theca and granulosa cells, which induces increasing and decreasing effects of FSH on estradiol and progesterone levels (27). In all of the three concentrations of sodium alginate, *Bmp4* gene expression level was more than 2D culture medium and the highest expression level of this gene was observed in concentration of 0.5%. 

*Bmp4*, as a paracrine growth factor, is secreted by theca and granulosa cells. *Bmp4*, along with *Bmp7*, is responsible for follicular growth regulation and primordial follicles transition to the primary form (28). West-Farrell et al. (28) examined expression levels of Bmp4 and *Bmp7* genes in sheep follicles. They observed that these two genes were not expressed in sheep follicles, while they were significantly expressed in mice follicles caused follicular development. Levels of gene expression of *Bmp4* and *Bmp7* were more in the fresh group compared to the vitrified group as well as the 3D culture compared to the 2D culture medium. Low expression of these genes in the follicle of 2D culture medium is different from the various concentrations of sodium alginate, because growth of the follicles depend on somatic and granulosa cells and any disorder of follicular cells causes a delay in preantral follicles development (29). In 2D culture of preantral follicles, granulosa cells disperse around the oocyte, resulting in a decreased relationship between oocyte and granulosa cells (30). 

The ovulation process, which is associated with molecular, cellular and biochemical changes, can lead to increase in the level of ROS. Physiological levels of ROS are essential for ovulation, normal function and success in conventional assisted reproductive technologies (14). *mnSOD* gene expression level in all of the three concentrations of sodium alginate was significantly higher than the control group. Among the different concentrations of sodium alginate, the highest and lowest expression levels of *mnSOD* gene were observed in 0.5% and 0.25% concentrations, respectively. Activation of transcription factors to enhance the antioxidant gene expression is a defense mechanism against free radical activity. The antioxidant enzymes are considered as the first defense line to metabolize toxic substances into harmless products. The first step in neutralizing the free oxygen radicals in the presence of superoxide dismutase (mnSOD) occurs in the mitochondria. Under the influence of SOD, superoxide anion is converted to H_2_ O_2_ with no radical activity but unfortunately it is changed rapidly to highly reactive hydroxyl radicals (31). Expression level of Gpx and Gcs genes in the all three concentrations of sodium alginate showed a significant increase compared to the control group. The highest expression level of this gene was related to the 0.5% concentration of sodium alginate. The first step in H_2_ O_2_ removal is the presence of antioxidant enzymes like glutathione peroxidase (GPX) and glutamyl cysteine synthase (GCS) in cytosol and mitochondria. These enzymes convert H_2_ O_2_ into _2_ H_2_ O. Combelles et al. (31) assessed the antioxidant genes in humans and mice. They found that only the Gcs gene was expressed in humans at the GV stage, but all of *Gpx, Gcs* and *mnSOD* genes were expressed in the MII phase. However, all of the mentioned genes were expressed in mice in both GV and MII phase.

Physical properties of the hydrogel are considered as the most critical factors affecting cell proliferation, growth factors, extracellular matrix and gene expression (32). As a result, the proper concentrations used in alginate hydrogel formation can provide the nutrient exchange, hormones and follicle expansion (33). In this study, the optimal concentration of alginate hydrogel for IVM was recognized at 0.5% concentration. The expression level of antioxidant genes in the fresh groups was higher than those of vitrified groups.

According to our results, level of the free radicals in both of the fresh and vitrified groups with 0.5% concentration showed significant decrease compared to the other concentrations and control group. Level of the free radicals in the fresh group with 0.5% concentration of sodium alginate and 2D culture medium was lower than the vitrified group. ROS has a dual function in culture medium; thus its certain level in IVM medium leads to the resumption of meiosis and maturation of the oocyte. Increased level of that is associated with a decreased level of follicle maturation and cessation of the cell cycle of egg cell. Thus, ROS in the IVM medium should be controlled in such a way to reduce the destructive effects (34). An inappropriate culture medium can reduce quality of the follicle. As it has been shown, developmental potential of the follicles is lower in the IVM medium than the follicle passing through the maturation stages *in vivo* conditions (35). In the culture medium, ROS production is also inevitable. Thus, the use of a culture medium with a concentration similar to the internal environment of the body can regulate the physiological levels of ROS (36, 37). According to the results of this study, the optimal concentration of sodium alginate to reduce the ROS level is found in 5%. This study showed that follicle maturation has several stages and the follicle vitrification with intrinsic potential property can damage the follicles. 

## Conclusion

The antifreeze agents used for the vitrification process could damage the cells by reacting with intracellular biomolecules and producing toxic substances. If these materials are used at high concentrations with enough time to contact with cells, their detrimental toxicity will appear causing cell destruction and death after the thawing step. But we used the method of incremental addition of the antifreeze to minimize its toxic effects and damages caused by increased cell quantity. Our results revealed that encapsulation of the follicles could conserve structure of the junctions between the cells and basement membrane of the granulosa cells, while preventing death of follicles.
